# Amnion Epithelial Cells Promote Lung Repair via Lipoxin A_4_

**DOI:** 10.5966/sctm.2016-0077

**Published:** 2016-11-07

**Authors:** Jean L. Tan, Yan Z. Tan, Ruth Muljadi, Siow T. Chan, Sin N. Lau, Joanne C. Mockler, Euan M. Wallace, Rebecca Lim

**Affiliations:** ^1^ The Ritchie Centre, Hudson Institute of Medical Research, Clayton, Victoria, Australia; ^2^ Department of Obstetrics and Gynecology, Monash University, Clayton, Victoria, Australia

**Keywords:** Lipoxin A_4_, Lung fibrosis, Inflammation, Macrophages, Neutrophils

## Abstract

Human amnion epithelial cells (hAECs) have been shown to possess potent immunomodulatory properties across a number of disease models. Recently, we reported that hAECs influence macrophage polarization and activity, and that this step was dependent on regulatory T cells. In this study, we aimed to assess the effects of hAEC‐derived proresolution lipoxin‐A_4_ (LXA4) on T‐cell, macrophage, and neutrophil phenotype and function during the acute phase of bleomycin‐induced lung injury. Using C57Bl6 mice, we administered 4 million hAECs intraperitoneally 24 hours after bleomycin challenge. Outcomes were measured at days 3, 5, and 7. hAEC administration resulted in significant changes to T‐cell, macrophage, dendritic cell, and monocyte/macrophage infiltration and phenotypes. Endogenous levels of lipoxygenases, LXA4, and the lipoxin receptor FPR2 were elevated in hAEC‐treated animals. Furthermore, we showed that the effects of hAECs on macrophage phagocytic activity and T‐cell suppression are LXA4 dependent, whereas the inhibition of neutrophil‐derived myleoperoxidase by hAECs is independent of LXA4. This study provides the first evidence that lipid‐based mediators contribute to the immunomodulatory effects of hAECs and further supports the growing body of evidence that LXA4 is proresolutionary in lung injury. This discovery of LXA4‐dependent communication between hAECs, macrophages, T cells, and neutrophils is important to the understanding of hAEC biodynamics and would be expected to inform future clinical applications. Stem Cells Translational Medicine *2017;6:1085–1095*

**Significance Statement**

In this study, stem‐like cells derived from the human placenta, called human amnion epithelial cells (hAECs), were shown to prevent lung injury by producing a lipid‐based molecule called lipoxin A_4_. This molecule works by encouraging interactions between a variety of immune cells in the lungs to effect repair. Following hAEC administration, fewer proinflammatory immune cells were attracted to the lungs and their activity was suppressed to achieve resolution of lung injury. The findings from this study will help inform the design of safe and efficacious hAEC therapy for lung disease.

## Introduction

Idiopathic pulmonary fibrosis (IPF) is a debilitating chronic inflammatory disease that affects more than 300 million people worldwide. The disease is characterized by progressive chronic inflammation; patients usually have limited numbers of CD4^+^/CD28^+^/CD25^+^/Foxp3^+^ regulatory T‐regulatory cells and higher numbers of Th1 effector CD4^+^ T cells in the lung [1]. Patients with severe forms of IPF also have significantly higher numbers of neutrophils, macrophages, and dendritic cells present in their bronchoalveolar lavage fluid (BALF) [2]. Currently, there is no effective treatment; lung transplantation is the only therapeutic option for end‐stage disease. However, cell‐based therapies have been suggested as a promising treatment approach [3, 4, 5, 6, 7, 8, 9]. Studies with early administration of mesenchymal stromal cells (MSCs) after bleomycin challenge reported significant anti‐inflammatory effects [10].

Preclinical studies have shown that stem cells reduce T‐cell proliferation, myeloperoxidase production, and profibrotic chemokine CCL‐2 and collagen deposition to repair lung injury [8, 11, 12]. Specifically, MSCs home to sites of injury and regulate inflammatory processes by reducing macrophage and neutrophil infiltration. These promising experimental studies led to early‐phase clinical trials of MSCs in patients with IPF, and these confirmed the safety of MSCs [13, 14, 15]. A number of efficacy trials of MSCs as a treatment for IPF are now underway [14]. However, if successful, the future application of MSCs is likely to be limited by the low cell yields from MSC harvesting. In that regard, human amnion epithelial cells (hAECs), stem‐like cells with potent immunomodulatory properties, are a promising alternative cell therapy. These cells appear equally effective in experimental models of lung injury [3, 5, 6, 7] and are more readily sourced in much greater cell numbers without the need for in vitro manipulation or expansion [4, 16, 17]. Like MSCs [18, 19], it would appear that the principle mechanisms of action of hAECs are paracrine, modulating the host immune cell responses and supporting the recruitment and expansion of host niche lung progenitor cells [20, 21, 22].

Although it is not essential to clinical application of hAECs, a better understanding of the paracrine signaling involved in hAEC‐modulated injury repair would be expected to improve the design of future clinical trials and, potentially, lead to novel non‐cell‐based therapeutics. In that regard, we wondered whether hAECs were exerting their reparative effects via lipoxins. Lipoxins are a subset of lipid mediators, inclusive of resolvins, protectins, and maresins, collectively called specialized proresolving mediators. Lipoxins are eicosanoids derived from arachidonic acid via enzymatic steps by lipoxygenases‐5, ‐12, and ‐15. They are regulators of inflammation and act by limiting polymorphonuclear neutrophil (PMN) infiltration and by enhancing nonphlogistic phagocytosis of apoptotic PMNs. Preclinical studies have shown the potent anti‐inflammatory properties of LXA4 in diverse disease models, including microbial infection, Alzheimer disease, liver transplantation, and myocardial ischemia reperfusion injury [23, 24, 25, 26]. In the lung, LXA4 and aspirin‐triggered lipoxin analogs have been shown to improve alveolarization in a neonatal model of hyperoxia‐induced injury and to prevent inflammation and fibrosis in bleomycin‐induced lung injury [27, 28, 29]. It has been shown that hAECs administered 24 hours after bleomycin challenge was beneficial [4, 8, 9]; therefore, we sought to assess the role of lipoxins in hAEC‐mediated lung repair in the same mouse model of lung injury.

## Materials and Methods

### Experimental Animals

Animal experiments were carried out with approval by Monash University Ethics Committee and conducted in accordance to the Australian Code of Practice for the Care and Use of Animals for Scientific Purposes. A total of 38 female mice, aged 8–12 weeks and weighing 18–21 g were administered 15 IU of bleomycin intranasally (Willow Pharmaceuticals, New South Wales, Australia http://willowpharma.com.au). Each mouse was randomly allocated to receive either 4 million hAECs in 0.2 ml of saline or saline alone via intraperitoneal injection 24 hours after receiving bleomycin. Mice were culled 1, 3, 5, or 7 days after bleomycin challenge. Bronchoalveolar lavage fluid and lung tissues were collected and processed as previously described [4].

### Human Amnion Epithelial Cell Isolation

Amnion cells were collected from placentae of consenting women undergoing an elective cesarean section delivery at term. Isolation of hAECs was performed according to previously described studies [16] in accordance with guidelines and approval from Monash Health Human Research Ethics Committee. Mean gestational age at collection was 38 weeks and 4 days. A total of 10 amnions were used in these in vitro and 1 of the 10 was randomly selected and used for all in vivo experiments.

### Tissue Collection

Animals were culled at days 1, 3, 5, and 7 by CO_2_ asphyxiation, and 2 ml of bronchoalveolar lavage fluid was collected with saline. The right lung was ligated at the right mainstem bronchus, then the trachea was exposed and the left lung was instilled with 4% paraformaldehyde. The right lung was excised for RNA analysis and the left lung for histological analysis. A separate cohort of animals was used for flow cytometric analysis, for which the entire lung (left and right) was processed (bleomycin and saline, *n* = 5, bleomycin and hAECs, *n* = 6).

### Histological and Immunohistochemical Analysis

#### Immunofluorescent Staining for Lipoxin Receptor and Macrophages

To measure the effect of hAECs on endogenous lipoxin A_4_ receptor expression and macrophage number, we performed immunohistochemistry for the lipoxin receptor N‐formyl peptide receptor 2 (FPR2) and for the macrophage marker F4/80 on lung slices. Briefly, paraffin‐embedded slices (0.5‐μm thick) were dewaxed and rehydrated in water. Antigen retrieval was performed with 10 mM citrate buffer, pH 6.0, in a microwave oven for 20 minutes. Blocking was performed with a universal protein blocking solution before immunostaining with anti‐FPR2 antibody at 1:100 (NSL1878; Novus Biologicals, Littleton, CO, https://www.novusbio.com) and anti‐F4/80 antibody at 1:200 (MCA497; Bio‐Rad Laboratories, Oxford, U.K., https://www.bio-rad-antibodies.com) with an overnight incubation at 4°C. Secondary antibody incubation was performed at room temperature for 1 hour, followed by nuclear stain with 4′,6‐diamidino‐2‐phenylindole (DAPI) for 10 minutes at room temperature. For each section, five fields of view were taken using the Nikon C1 confocal microscope running the NIS Elements Software (Nikon, Tokyo, Japan, http://www.nikon.com), where dual positive staining was manually quantified and analyzed with FIJI ImageJ analysis software (version 1.480; http://imagej.net/).

### Flow Cytometry

Whole lungs were perfused with saline and minced using a tissue chopper (Campden Instruments, Lafayette, IN, http://campdeninstruments.com). Lung tissues were digested in Dulbecco’s modified Eagle’s medium‐F12 media (11330‐057; Thermo Fisher Scientific Life Sciences, Waltham, MA, http://www.thermofisher.com) containing 25 mg/ml collagenase IA (10103578001; Roche, NSW, Australia, http://www.roche.com), 2.5 mg/ml DNase I (AMPD1; Sigma‐Aldrich, St. Louis, MO, http://www.sigmaaldrich.com), and 10% (volume per volume) heat‐inactivated fetal bovine serum (16110‐082; Thermo Fisher) for 15 minutes at 37°C. Lung lysates were passed through a 70‐μM cell strainer and red blood cells were lysed. Fc receptors were blocked with anti‐CD16/32 (BD Biosciences, San Jose, CA, http://www.bdbiosciences.com) before staining for CD45, CD4, CD11b, F4/80, Ly6C, and CD11c. Data were acquired using a BD LSR II analyzer (BD, Franklin Lakes, NJ, http://www.bd.com). Representative gating strategies are shown in supplemental online Figure 1.

### Neutralization of Lipoxygenases With Nordihydroguaiaretic Acid in hAECs

Human amnion epithelial cells were cultured in a T75 flask at a density of 5 × 10^6^ cells. Neutralization of lipoxygenases was performed by adding 2.5 µM or 10 µM of nordihydroguaiaretic acid (NDGA) and incubated at 37°C for 24 hours. After the 24‐hour incubation, hAECs were collected for quantitative polymerase chain reaction analysis of lipoxygenase‐5, ‐12, and ‐15 expression and supernatant was collected for enzyme‐linked immunosorbent assay (ELISA) of LXA4 before further coculture studies. Conditioned medium was obtained according to previous protocols after neutralization of NDGA [18].

### Macrophage Phagocytic Assay

Macrophage phagocytosis was determined as previously described [18]. Briefly, macrophages were plated in 6‐well flat‐bottom culture plates at a density of 5 × 10^5^ cells per well for 48 stimulated lipopolysaccharide (LPS; 10 ng/ml) with or without NDGA preprimed hAECs and with or without primary hAECs (1:1 ratio). *Staphylococcus aureus* particles labeled with pHrodo (Thermo Fisher) were added to each well (10 μg/ml) and incubated for 30 minutes. Incubation on ice inhibits membrane movement and was used as a negative control. Only cells that phagocytosed pHrodo‐labeled *S. aureus* were fluorescent and stained positive on fluorescence‐activated cell sorting (FACS).

### Measuring T‐Cell Proliferation and Migration

Naïve T cells were isolated from spleens of C57Bl/6 mice using a CD4 magnetic‐bead isolation kit (130‐095‐248; Miltenyi Biotec, San Diego, CA, http://www.miltenyibiotec.com). CD4‐enriched T cells (0.5 × 10^6^) labeled with carboxyfluorescein succinimidyl ester were stimulated with CD3ε at 10 μg/ml (MAB484; R&D systems, Minneapolis, MN, https://www.rndsystems.com) and 2 μg/ml CD28 (553294; BD Biosciences) in complete Roswell Park Memorial Institute (RPMI) medium with or without NDGA preprimed hAECs and with or without primary hAECs (ratios: 1:2, 1:5, 1:10). T cells were cocultured directly with hAECs for 72 hours before staining with SYTOXBlue dead‐cell dye (1 μM; S34857; Thermo Fisher) and analyzed by flow cytometry (LSRII analyzer; BD). For T‐cell migration, enriched CD4^+^ cells were added to the top chamber of a transwell insert in a 24‐well plate and stimulated with CCL17 in either complete RPMI medium or hAEC‐conditioned medium. T cells that migrated across the transwell membrane were counted after 4 hours.

### Neutrophil Myeloperoxidase Activity Assay

Neutrophils were isolated from mouse femoral bone‐marrow exudates using a CD11b magnetic‐bead isolation kit (Miltenyi Biotec). Enriched neutrophils (0.5 × 10^6^) were cultured in complete RPMI with or without NDGA‐preprimed hAECs and with or without primary hAECs (0.5 × 10^6^), and activated with granulocyte‐colony stimulating factor (100 ng/ml; Amgen, Sydney, Australia, http://www.amgen.com) for 12 days. The medium was changed every other day. After 12 days, cells were lysed with cetyltrimethylammonium bromide at 500 μl per 1 × 10^6^ cells. Then, 50 μl was added to 950 μl of 50 mM potassium phosphate buffer (pH 6.0) that contained 0.167 mg/ml *o*‐dianisidine (D9143; Sigma‐Aldrich) and 0.0005% H_2_O_2_ (Sigma‐Aldrich), and the absorbance at 460 nm (A460) was measured. One unit of myeloperoxidase (MPO) activity was defined as a change in A460 of 1.0 after 5 minutes; results were expressed as mU of MPO activity per milligram of lysate.

### LXA4 ELISA or Th1/Th2/Th17 Cytokine Bead Array

The concentration of lipoxin A_4_ in BALF and Th1/Th2/Th17 cytokines in culture supernatants were measured by ELISA and bead array according to manufacturer’s guidelines. Data were collected using the FACS LSRII analyzer and analyzed with BD FRAP Array software (BD Biosciences).

### Statistical Analysis

Data were expressed for each experimental group as mean ± SEM. Differences between two experimental groups were determined using an unpaired, one‐tailed *t* test. Differences across three or more experimental groups were determined using one‐way analysis of variance with the Bonferroni post hoc test. Confidence intervals of 95% were deemed significant. All analyses were performed using GraphPad Prism (GraphPad Software, San Diego, CA, http://www.graphpad.com).

## Results

### Effects of hAECs on Cytokines and Inflammatory Cells

**Figure 1:**
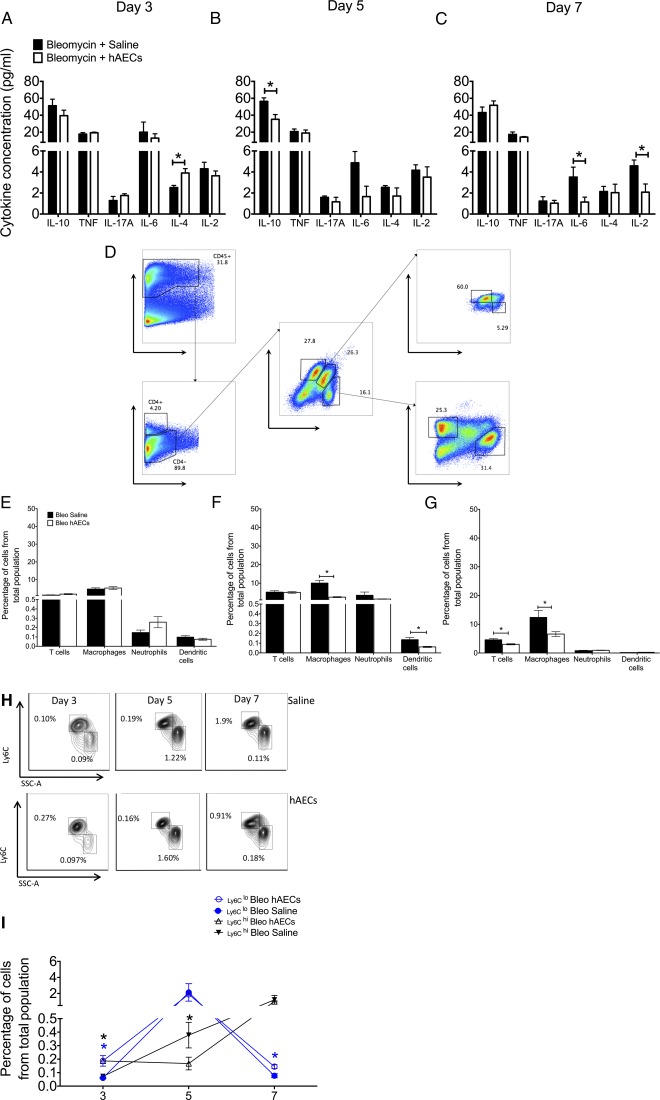
hAECs altered cytokine expression and immune cell infiltrate in vivo. **(A):** Administration of hAECs significantly increased anti‐inflammatory IL‐4 cytokine levels at the onset of inflammation (control group: 2.52% ± 0.20% vs. hAEC group: 3.90% ± 0.42%). Expression of IL‐10 **(B)** and inflammatory IL‐6 and IL‐2 levels **(C)** were reduced in animals that received hAECs (control vs. hAEC groups: IL‐10: 56.36% ± 4.199% vs. 35.17% ± 5.67%; IL‐6: 3.52% ± 0.94% vs. 1.14% ± 0.46%; IL‐2: 4.58% ± 0.55% vs. 2.08% ± 0.78%). **(D):** Immune cell populations were measured via flow cytometry with gating strategies. Percentages on flow cytometry plots represent the percentages from the parent population. Table 1 lists markers used for each population. There was no change in percentage in all immune cell types at day 3 (E). **(F, G):** F4/80‐positive macrophage numbers were reduced by day 5 **(F)** and day 7 **(G)** in mice given hAECs (day 5: 10.05% ± 1.48% vs. 2.49% ± 0.25%; day 7: 12.39% ± 2.45% vs. 6.59% ± 0.84%). **(F):** Dendritic CD11c+ cell numbers were significantly reduced at day 5 (control group: 0.13% ± 0.02% vs. hAEC group: 0.06% ± 0.01%). **(G):** Administration of hAECs slightly reduced CD4 T‐cell numbers in the lung at day 7 (control group: 4.60% ± 0.40% vs. hAEC group: 3.03% ± 0.20%). **(H):** Representative flow cytometry plots for monocytic subpopulations CD11b+/ Ly6C+ cells are shown. Percentages on flow cytometry plots represent the percentages from parent population. **(I** **):** Changes in Ly6C^lo^ and Ly6C^hi^ cells across days 3, 5, and 7 are shown. Percentage of Ly6C^hi^ cells peaked at day 5 and decreased by day 7 in both groups; hAEC administration resulted in significantly increased percentages of Ly6C^lo^ monocytes at days 3 and 7 (day 3: 0.072% ± 0.012% vs. 0.187% ± 0.039%; day 7: 0.084% ± 0.013% vs. 0.145% ± 0.015%). in both groups. The percentage of Ly6C^hi^ cells gradually increased from day 3 to day 7 in both groups. hAEC treatment resulted in significantly increased percentages of Ly6C^hi^ cells on days 3 and 5 (day 3: 0.001% ± 0.0002% vs. 0.005% ± 0.001%; day 7: 0.006% ± 0.001% vs. 0.053% ± 0.011%). ∗, *p* < .05. Abbreviations: bleo, bleomycin; hAEC, human amnion epithelial cell; IL, interleukin; TNF, tumor necrosis factor; FSC, forward scatter; SSC, side scatter.

hAEC administration significantly increased IL‐4 expression in the lung at day 3 (2.52 ± 0.20 pg/ml vs. 3.90 ± 0.42 pg/ml, *p* = .012; Fig. 1A) and significantly decreased levels of IL‐10 at day 5 (53.36 ± 4.20 pg/ml vs. 35.17 ± 5.67 pg/ml, *p* = .015; Fig. 1B) and of IL‐6 and IL‐2 at day 7 (IL‐6: 3.52 ± 0.95 pg/ml vs. 1.14 ± 0.46 pg/ml, *p* = .048; IL‐2: 4.58 ± 0.56 pg/ml vs. 2.08 ± 0.78 pg/ml, *p* = .026; Fig. 1C).

Next, we assessed changes to the immune‐cell population in the lung based on surface markers listed in Table 1 and gated according to the gating strategies seen in Figure 1D.

**Table 1:** Characterization of immune cell types

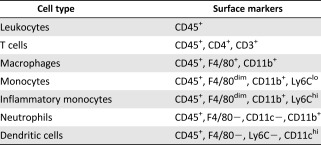

There were no significant changes in percentages of all cell types at day 3 (Fig. 1E). At day 5, the percentage of macrophage and dendritic cells, compared with that of bleomycin controls, was significantly lower in hAEC‐treated animals (macrophages: 10.054% ± 1.483% vs. 2.496% ± 0.249%, *p* = .001; dendritic cells: 0.136% ± 0.022% vs. 0.061% ± 0.006%, *p* = .012; Fig. 1F). At day 7, compared with control mice, administration of hAECs decreased percentages of CD4+ T cells and macrophages (T cells: 4.604% ± 0.404% vs. 3.026% ± 0.204%, *p* = .008; macrophages: 12.394% ± 2.452% vs. 6.597% ± 0.838%, *p* = .039; Fig. 1G). Figure 1H is a representative flow cytometry plot of F4/80^lo^/CD11b^+^/Ly6C^hi^/SSC‐A^lo^ cells. Taken together, these findings demonstrate that hAEC treatment 24 hours after bleomycin exposure suppressed the macrophage and dendritic cell milieu during onset of inflammation. Subsequently, inflammatory T‐cell infiltration is restricted either through hAEC signaling or from lack of macrophage and dendritic cell signaling.

No significant differences in percentage of T‐cell, neutrophil, macrophage, and dendritic cells between any groups were observed at day 3. However, when we looked closer into subpopulations of inflammatory monocytes/macrophages, compared with the bleomycin control group, animals treated with hAECs had a significantly higher percentage of Ly6C^hi^ monocytes at days 3 and 7 (day 3: 0.072% ± 0.012% vs. 0.187% ± 0.039%; day 7: 0.084% ± 0.013% vs. 0.145% ± 0.015%; *p* < .05 for both; Fig. 1I).

### hAECs’ Effect on Lipoxin A_4_ and its Receptor, FPR2

**Figure 2:**
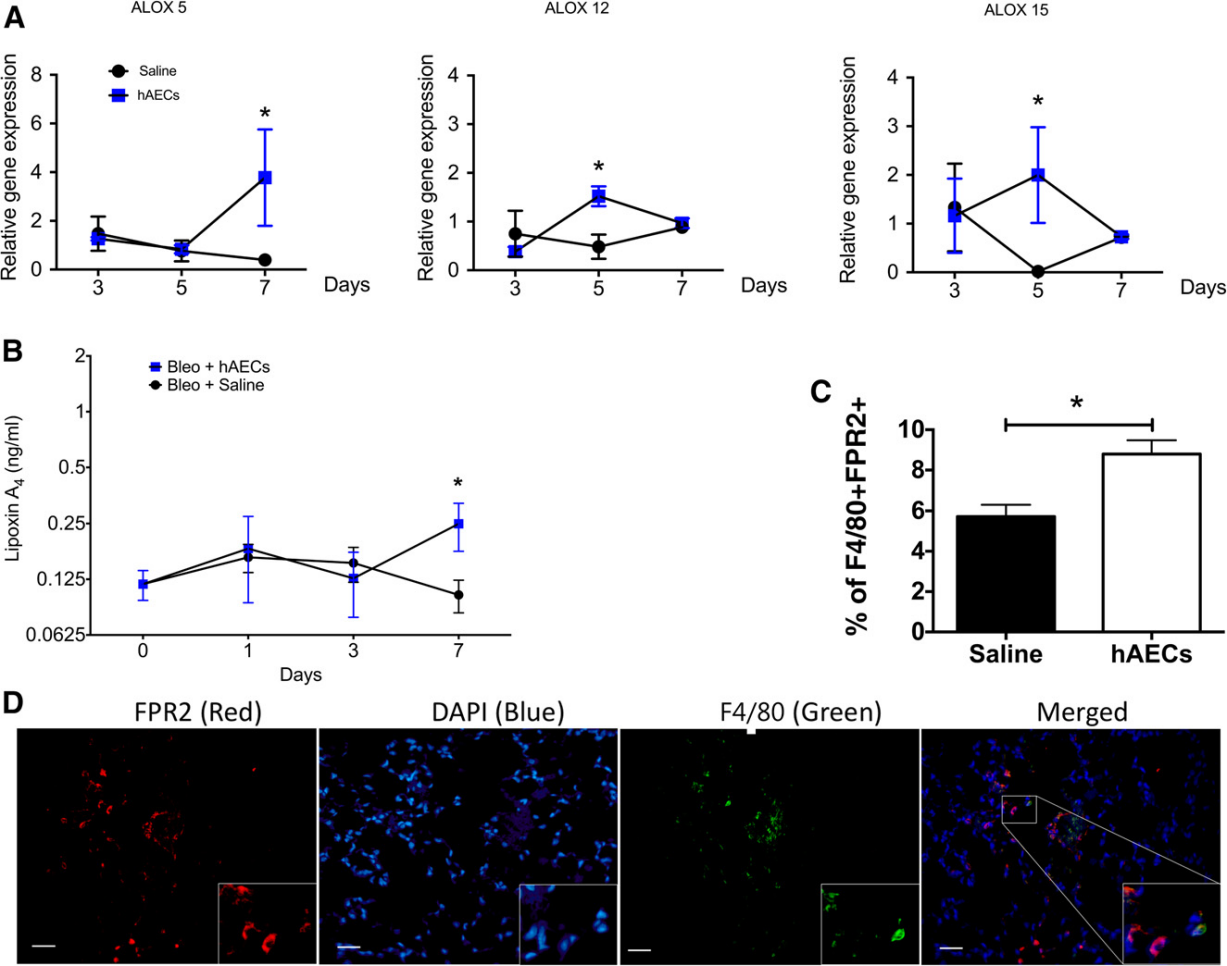
Lipoxin A_4_, along with its precursors and ligand, were altered by hAECs. **(A):** Expression of the lipoxin A_4_ precursor genes *ALOX‐5, ‐12*, and *‐15* was increased at days 5 and 7 compared with saline controls (*ALOX‐5:* 0.393 ± 0.114 vs. 3.777 ± 1.98; *ALOX‐12:* 0.484 ± 0.248 vs. 1.520 ± 0.203; *ALOX‐15:* 0.020 ± 0.007 vs. 1.998 ± 0.983). ∗, *p* < .05. **(B):** Lipoxin A_4_ protein levels were elevated in lung lysates at day 7 in animals treated with hAECs compared with controls (0.103 ± 0.021 ng/ml vs. 0.249 ± 0.072 ng/ml, respectively). ∗, *p* < .05. **(C):** At day 7, bleomycin challenge resulted in positively stained F4/80/FPR2 cells, which were was elevated in mice treated with hAECs compared with control mice (5.720% ± 0.587% vs. 8.795% ± 0.687%, respectively). ∗, *p* < .05. (D): Representative images of F4/80‐ and FPR2‐positive‐stained lung sections from hAEC‐treated animals. Magnification: ×200. Scale bar =100 μm. Abbreviations: DAPI, 4′,6‐diamidino‐2‐phenylindole; FPR2, *N*‐formyl peptide receptor 2; hAEC, human amnion epithelial cell.

Given earlier reports that lipoxin A_4_ is highly expressed in placental tissues [20, 22] and plays a valuable role in resolving inflammation and fibrosis, we evaluated the role of lipoxin A_4_ in hAEC‐mediated lung repair. Protein analysis of bronchoalveolar lavage fluids revealed that, compared with the control group of animals treated with bleomycin plus saline, hAEC administration was associated with significantly higher lipoxin A_4_ levels at day 7 (0.13 ± 0.02 vs. 0.25 ± 0.07; *p* = .036; Fig. 2B) despite no changes in total protein content (supplemental online Fig. 1). This was accompanied by a significant increase in the gene expression of lipoxygenase‐5 on day 7, lipoxygenase‐12 and ‐15 on day 5 (*ALOX‐5*: 0.393 ± 0.114 vs. 3.78 ± 1.98, *p* = .013; *ALOX‐12*: 0.484 ± 0.248 vs. 1.52 ± 0.20, *p* = .018; *ALOX‐15*: 0.02 ± 0.01 vs. 1.99 ± 0.98, *p* = .019; Fig. 2A). Compared with the control group, hAEC treatment was associated with significantly elevated expression of F4/80 and FPR2 in the lungs of day 7 animals (Fig. 2C). Representative immunofluorescent staining showing colocalization positive for the macrophage marker F4/80, FPR2, and nuclear stain DAPI of hAEC‐treated lung is shown in Figure 2D.

### Effect of hAECs on Macrophage and Neutrophil Function

**Figure 3:**
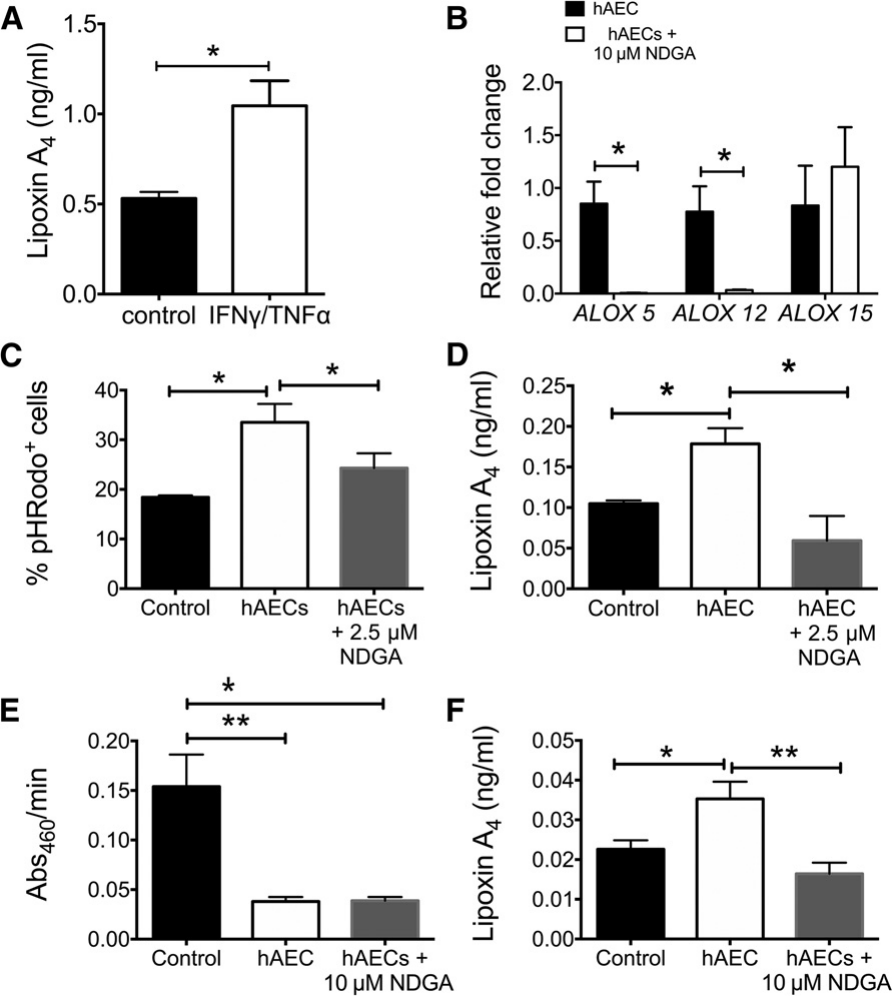
Lipoxin A_4_ is required for hAEC‐mediated upregulation of macrophage phagocytosis. **(A):** hAECs expressed elevated lipoxin A_4_ in the presence of inflammatory cytokines IFN‐γ and TNF‐α (control group: 0.531 ± 0.036 ng/ml vs. IFN‐γ/TNF‐α: 1.045 ± 0.139 ng/ml). ∗, *p* < .05. **(B):** Pretreating hAECs with NDGA significantly reduced its precursors ALOX‐5 and ‐12 compared with hAEC alone (ALOX‐5: 0.852 ± 0.209 vs. 0.004 ± 0.002, ALOX‐12: 0.776 ± 0.241 vs. 0.032 ± 0.005, respectively). **(C):** Bone‐marrow‐derived macrophages cultured with hAECs demonstrated increased uptake of pHrodo‐labeled bacterial particles in comparison with control medium. Preculturing hAECs with lipoxygenase inhibitor NDGA reversed macrophage phagocytic uptake of pHrodo particles (control group: 18.40% ± 0.374% vs. hAEC group: 33.52% ± 3.703% vs. hAEC plus NDGA group: 24.29% ± 2.985%). ∗, *p* < .05. **(D):** Concentration of LXA4 in the culture supernatant was measured. Protein level of LXA4 increased in the presence of hAECs and was reduced when hAECs were precultured with NDGA (control group: 0.105 ng/ml ± 0.004 ng/ml vs. hAEC group: 0.178 ng/ml ± 0.019 ng/ml vs. hAEC plus NDGA group: 0.059 ng/ml ± 0.030 ng/ml). ∗, *p* < .05; ∗∗∗, *p* < .001. **(E):** Neutrophil myeloperoxidase activity in the presence of hAECs was significantly reduced and, conversely, addition of NDGA did not alter neutrophil activity (absorbance at 460 nm/minute: control group: 0.154 ± 0.032 vs. hAEC group: 0.038 ± 0.005 vs. hAEC plus NDGA group: 0.038 ± 0.004). ∗∗, *p* < .01. **(F):** Culturing neutrophils with NDGA‐precultured hAECs significantly reduced LXA4 protein synthesis (control group: 0.02 ± 0.002 ng/ml vs. hAEC group: 0.04 ± 0.004 ng/ml vs. 0.01 ± 0.002 ng/ml). ∗, *p* < .01; ∗∗, *p* < .05. Abbreviations: Abs, absorbance; hAEC, human amnion epithelial cell; IFN, interferon; NDGA, nordihydroguaiaretic acid; TNF, tumor necrosis factor.

Pulmonary inflammation induced by bleomycin is managed through a balance of neutrophil infiltration, apoptosis, and subsequent macrophage efferocytosis of apoptotic neutrophils. Lipid mediators, in particular, LXA4, are potent regulators of this process and, accordingly, we evaluated the direct effects of hAEC‐derived LXA4 on macrophage and neutrophil function in vitro. We first showed that we could induce and inhibit hAEC production of LXA4 and its precursor enzymes ALOX‐5, ‐12, ‐15. Proinflammatory cytokines interferon (IFN)‐γ and tumor necrosis factor (TNF)‐α significantly increased LXA4 production in hAECs (0.5309 ± 0.0359 ng/ml vs. 1.0450 ± 0.1393 ng/ml, *p* = .012; Fig. 3A).

Next we showed that in the presence of nordihydroguaruretic acid (NDGA), gene expression of lipoxygenase‐5 and ‐12 was significantly reduced as well (*ALOX*‐5: 0.852 ± 0.209 vs. 0.004 ± 0.002, *p* = .004; *ALOX‐12*; 0.776 ± 0.241 vs. 0.032 ± 0.005, *p* = .015; *ALOX‐15*; 0.834 ± 1.200 ± 0.374; Fig. 3B), suggesting that biosynthesis of LXA4 may occur in hAECs in an inflammatory environment.

Using bone‐marrow‐derived macrophages, we showed that hAECs increased phagocytic activity as previously described [18], and this effect was diminished with the addition of 2.5 µM NDGA (data not shown for 10µM; respective data for control, hAEC, and hAEC plus NGDA groups: 18.40% ± 0.374% vs. 33.52% ± 3.703% vs. 24.29% ± 2.985%, *p* = .0313; Fig. 3C). We also confirmed that NDGA successfully inhibited LXA4 production by hAECs in macrophage cultures for the three groups (0.105 ± 0.004 ng/ml vs. 0.178 ± 0.019 ng/ml vs. 0.116 ± 0.043 ng/ml [*p* = .059 and *p* = .0317, respectively]; Fig. 3D).

Next, we assessed the effects of hAECs on myeloperoxidase (MPO) production by primary neutrophils. Whereas coculture of CD11b^+^ neutrophils with hAECs significantly reduced their production of MPO, the addition of 2.5 µM (data not shown) and 10 μM NDGA did not affect MPO production, as determined by absorbance measurements (0.154 ± 0.0323 A460/minute vs. 0.038 ± 0.005 A460/minute vs. 0.039 ± 0.004 A460/minute, *p* = .001; Fig. 3E). Successful inhibition of hAEC‐derived lipoxin A_4_ by NDGA is shown in Figure 3F (0.023 ± 0.002 ng/ml vs. 0.035 ± 0.004 ng/ml vs. 0.016 ± 0.003 ng/ml [*p* = .037 and *p* = .005, respectively]; Fig. 3F).

### hAEC‐Mediated Change in T‐Cell Phenotype Is Not Mediated via Lipoxin A_4_

**Figure 4:**
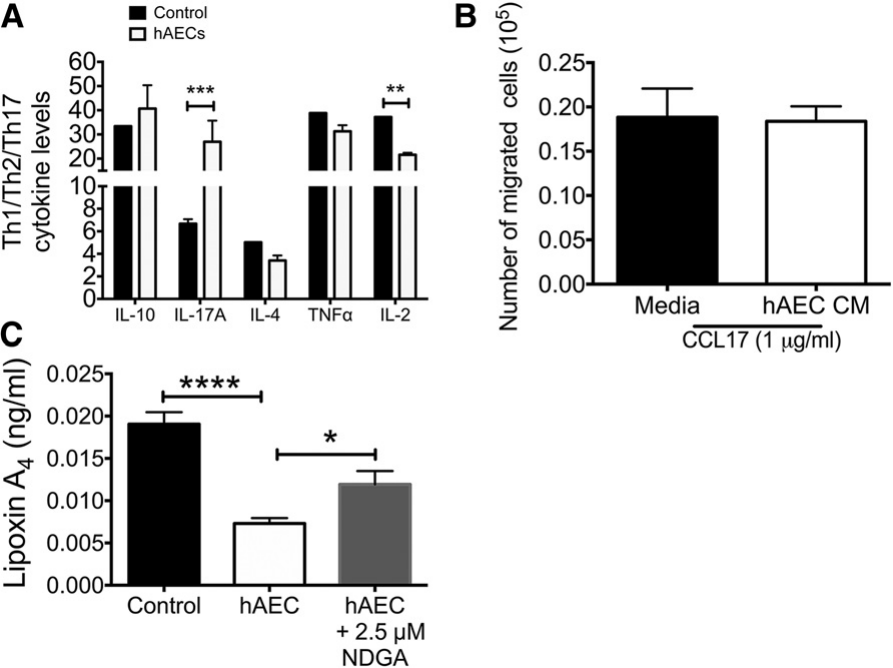
hAECs alter T‐cell proliferation through cytokines. **(A):** CD4^+^ T cells enriched from splenocytes were cultured with hAECs, and the supernatant Th1/Th2/Th2 cytokine profile was measured. In the presence of hAEC, naïve T cells expressed significantly more IL‐17A but less IL‐2 (IL‐17A: 6.67 ± 0.40 vs. 26.96 ± 8.76; IL‐2: 37.20 ± 0.00 vs. 21.60 ± 0.81). ∗∗, *p* < .01; ∗∗∗, *p* < .001. **(B):** We then evaluated the effect of hAECs on T‐cell behavior by supplementing cultures with either chemokine CCL17 or CD3ε/CD28 to assess migration and proliferation, respectively. In the presence of CCL17, T cells increased motility; however, this effect was not attenuated by hAECs (medium: 0.19 ± 0.033 cells vs. hAEC CM: 0.18 ± 0.017 cells). **(C):** T‐cell proliferation was significantly suppressed by presence of hAECs after 72 hours in culture (Proliferation Index score: control group: 1.508 ± 0.023 vs. hAEC group: 1.077 ± 0.018 vs. hAEC plus NDGA group: 1.57 ± 0.098). ∗, *p* < .05. Inhibiting lipoxygenases with NDGA significantly attenuated hAEC‐mediated suppression of T‐cell proliferation **(C)** but not LXA4 levels (data not shown). Abbreviations: CCL, chemokine (C‐C motif) ligand; CM, conditioned medium; hAEC, human amnion epithelial cell; IL, interleukin; NDGA, nordihydroguaiaretic acid; TNF, tumor necrosis factor.

We evaluated the effect of hAECs and hAEC‐derived LXA4 on T‐cell phenotype, function, and behavior. In comparison with the control treatment (i.e., medium alone), hAECs significantly decreased IL‐2 and increased IL‐17A levels in vitro (IL‐2: 37.20 ± 0.00 vs. 21.60 ± 0.81, *p* = .031; IL‐17A: 6.670 ± 0.40 vs. 26.960 ± 8.76, *p* = .005; Fig. 4A). We showed that T‐cell chemotaxis toward CCL17 was unaltered by hAECs (Fig. 4B). However, coculture with hAECs significantly suppressed T‐cell proliferation, which diminished in the presence of 2.5 µM NDGA (data not shown for 10 µM; 1.508 ± 0.024 vs. 1.077 ± 0.018 vs. 1.570 ± 0.098 [*p* = .006 and *p* = .001, respectively]; Fig. 4C).

## Discussion

Owing to their extensive immunomodulatory properties, stem cells and stem‐like cells such as hAECs have been suggested as therapeutic avenues for treating IPF. This is supported by a study showing the role of lipoxins in cell therapy‐mediated lung repair, where human bone‐marrow‐derived MSCs induced production of LXA4 by alveolar epithelial cells in a mouse model of LPS‐induced acute lung injury [30]. Guilherme and colleagues showed that LXA4 analogs were potent anti‐inflammatory and antifibrotic compounds in a bleomycin‐induced model of lung injury [27]. We thus focused our attention on lipoxin A_4_ a key regulator of macrophage infiltration and function in hAEC‐mediated repair of lung fibrosis.

We observed that hAEC treatment was associated with increased IL‐4 cytokine expression at day 3 after bleomycin challenge, accompanied by a reduction in IL‐10 at day 5, and IL‐6 and IL‐2 at day 7. Levels of inflammatory cytokines may affect immune cell infiltration into the lung, as we observed increased inflammatory monocyte numbers at day 3 followed by fewer macrophages and dendritic cells by day 5. These changes progressed, and on day 7, CD4^+^‐naïve T‐cell and macrophage numbers were reduced in animals treated with hAECs. Next, we measured the effect of hAEC administration on endogenous production of lipoxin A_4_ and its receptor FPR2, and showed significant increase in LXA4 levels in BALF and increased macrophage FPR2 expression on lung tissue at day 7. We further characterized lipoxygenase‐mediated effects of hAECs on neutrophil, macrophage, and T‐cell behavior, using a lipoxygenase‐neutralizing compound, NDGA. We showed that hAECs increased macrophage phagocytosis and suppressed T‐cell proliferation in a lipoxygenase‐mediated fashion. However, hAECs’ ability to reduce primary neutrophil MPO activity appeared to be independent of LXA4.

Polarity of Th1 and Th2 cells is a stochastic process usually directed by proinflammatory (i.e., IL‐6, IL‐2, IL‐12) and anti‐inflammatory (i.e., IL‐10, IL‐4) cytokines, which provides positive feedback signaling for downstream inflammatory response. Bronchoepithelial cells express IL‐4 receptors, which, upon activation by IL‐4, results in the production of cytokines (i.e., monocyte‐chemotactic protein ‐1, IL‐8, and IL‐1Ra) and increased migration of human airway epithelial cells [31, 32]. IL‐4 is a known profibrotic cytokine in preclinical studies using bleomycin. Huaux and colleagues showed that IL‐4^−/−^ mice have lower profibrotic factors such as OH‐proline, soluble collagen, fibronectin, and tumor growth factor (TGF)‐β1 from day 14 onward [33]. However, as our current study investigates changes during acute inflammation, we draw our attention toward the role of IL‐4 in inflammation. Interestingly, IL‐4 is a potent inducer of lipoxygenase ‐12 and ‐15. Chaitidis et al. showed that human peripheral blood monocytes strongly upregulated LOX‐15 and conversely downregulated classical proinflammatory cytokines TNFα, IL‐6, IL‐1, and IL‐8 when cultured with IL‐4 [34]. In our study, we observed that administration of hAECs modestly increased levels of IL‐4 at day 3. This suggests that application of hAECs can be detrimental in a fibrotic environment but beneficial in an inflammatory environment, and considerations toward clinical applications should be made with this in mind.

Conversely, we found significant reduction in Th1 drivers IL‐6 and IL‐2 by day 7, suggesting a repression of inflammatory responses. This is supported by an earlier report that showed blocking IL‐6 at days 8, 9, and 10 after bleomycin instillation can ameliorate lung fibrosis [35]. Surprisingly, we observed a transient reduction in IL‐10 levels on day 5, which contradicts a previous finding by Manuelpillai and colleagues that described an increase in IL‐10 levels following hAEC treatment in a mouse model of liver fibrosis [36]. CD11c dendritic cells [37, 38] and macrophages are potent producers of IL‐10. Sakamoto and colleagues showed that after bleomycin challenge, the levels of IL‐10 in bronchoalveolar mononuclear cells began increasing at day 3, then peaked at day 7 and persisted until day 14. It is, therefore, likely that the reduced levels of IL‐10 on day 5 are reflective of the reduced infiltration of dendritic cells and macrophages on day 3 [39].

Lipoxin A_4_ is a lipid mediator capable of anti‐inflammatory signaling by regulating neutrophil infiltration and proresolving signaling through macrophage polarization and nonphlogistic uptake of apoptotic PMNs. Their innate ability to perpetuate and limit inflammation is of particular importance in our study to define probable immune regulatory roles of hAECs. As our first step in identifying the importance of LXA4 in hAEC‐mediated repair, we evaluated endogenous levels of LXA4 and gene expression of its precursor enzymes ALOX‐5, ‐12, and ‐15 in vivo. Here, hAECs significantly increased levels of LXA4 in BALF at day 7 after bleomycin challenge. Gene expression of the enzymes ALOX‐5, ‐12, and ‐15 responsible for the biosynthesis of lipoxin was significantly increased in hAEC‐treated animals at day 7 (ALOX‐5) and day 5 (ALOX‐12 and ‐15), suggesting that hAEC treatment had an effect on endogenous LXA4 and lipoxygenase synthesis in bleomycin‐induced lung injury. However, changes to lipoxygenase expression varied between days 5 and 7 and may be a consequence of the behavior of lipoxygenase‐expressing immune cells [40]. Treatment with hAECs could indirectly contribute to lipoxygenase expression of endogenous immune cells.

In our study, we saw no change in neutrophil numbers across all days between injured and treated animals, suggesting that, in our study at least, hAECs did not alter neutrophil infiltration to the site of injury and may, instead, mediate activity of neutrophils to resolve injury. Neutrophils are cells recruited as the first line of inflammatory defense, hence behavioral changes to these cells will inevitably influence downstream inflammatory processes. Neutrophil release of MPOs contributes to alveolar damage and, subsequently, fibrosis in patients with IPF, and their activity can be regulated by aspirin‐triggered 15‐*epi*‐lipoxin A_4_ in vitro [41, 42, 43]. Our previous studies showed that hAECs significantly reduced proinflammatory MPO‐positive cells in vivo [4, 8]. In this study, we showed that hAECs reduced neutrophil MPO activity in vitro, but this was unlikely to be mediated through LXA4. Although these results are contrary to our initial hypothesis, it was unsurprising, because other lipid‐based mediators are involved in this process. Resolvin E1 administration inhibited PMN infiltration and reduced MPO levels in aspiration pneumonia in mice [44, 45]. Taken together, these studies suggest that hAEC inhibition of neutrophil MPO activity is driven by alternatives to the lipoxygenase pathway.

Next, we assessed the changes to pulmonary monocyte subpopulations where two distinct subtypes can be found: Ly6C^hi^ monocytes, which secrete inflammatory cytokines TNF‐α and IL‐1β; and Ly6C^lo^ monocytes, which patrol the endothelium. The percentage of CD11b^+^Ly6C^hi^ monocytes was significantly higher in hAEC‐treated animals at days 3 and 5 of this study. This was unexpected because there was less inflammation and honeycomb injury in those mice. Although denoted as inflammatory monocytes, CD11b^+^Ly6C^hi^ cells have the ability to differentiate into mature alternatively activated macrophages [46]. Gibbons and colleagues reported that the adoptive transfer of bone‐marrow‐derived Ly6C^hi^ inflammatory monocytes during the progressive fibrotic phase led to increased numbers of M2 macrophages and exacerbation of fibrosis [47, 48, 49]. In a similar vein, M2 polarization during the peak of lung inflammation has been reported to have a protective effect in the bleomycin model [18], an outcome that was not achieved with later administration of hAECs, coinciding with the progressive fibrotic phase [45, 50]. Collectively, this suggests that the spatiotemporal infiltration of inflammatory monocytes could be supporting an M2‐driven proreparative response at day 7. Additionally, increase in LXA4 levels at day 7 in animals treated with hAECs could lead to the increase in inflammatory monocytes seen in our study. Studies have shown that LXA4 stimulates monocyte migration, adherence, and Ca^2+^ motility [51, 52]. This finding coincided with increased numbers of FPR2+ macrophages in lung tissues at day 7 in mice treated with hAECs; however, FPR expression was not wholly restricted to macrophages. This is unsurprising because FPR2 expression is not restricted to macrophages and is expressed by other phagocytic leukocytes such as neutrophils. Given that annexin A1 has been shown to bind strongly to FPR2 on the surface of activated PMNs [53] and is critical for leukocyte adhesion and rolling, as well as the attenuation of MPO activity in neutrophils [54], FPR2‐annexin A1 interaction could explain the nonmacrophage effects observed in our current study.

Our observations suggest that hAECs exert their effects, at least in part, through LXA4, which is a potent stimulus for macrophage phagocytosis [55, 56]. Macrophage phagocytosis of apoptotic PMNs is a rate‐limiting step in inflammation, which could contribute to the pathogenesis of IPF. Alveolar macrophages of patients with IPF have reportedly impaired efferocytosis activity compared with alveolar macrophages from patients affected by other forms of interstitial lung disease [57, 58]. It is plausible, therefore, that hAECs’ ability to promote phagocytosis may contribute toward lung repair. However, to determine if hAEC‐mediated increase in phagocytosis was driven by LXA4, we showed that neutralization of lipoxygenases negated the ability of hAECs to promote macrophage phagocytosis. Data reported here extend previous observations that hAECs polarized macrophages from an M1 to an M2 phenotype by increasing their mannose receptor CD206 expression [18]. As shown by Shirey and colleagues, neutralization of lipoxygenase pathways inhibits M2 macrophage differentiation in mice infected with respiratory syncytial virus [50, 59]. Moreover, Maderna and colleagues showed that bone‐marrow‐derived macrophages from *fpr2^−/−^* have defective phagocytic ability for apoptotic PMN [25, 60], further supporting the importance of LXA4‐FPR2 binding in regulating macrophage phagocytosis.

LXA4 is a potent regulator of inflammation and resolution; however, other specialized lipid mediators released by hAECs remain to be investigated [61, 62, 63]. Our study showed an increase in LXA4 levels in vivo and in vitro, albeit at low levels (<0.05 ng/ml). Notably, lipoxins are formed in picogram and nanogram amounts in humans, with their potency maintained. Guilherme and colleagues showed that treatment with relatively low concentrations of 1 μg and 0.1 μg of aspirin‐triggered lipoxin A_4_ per mouse at days 7 and 10 significantly reduced inflammatory cell infiltration and reversed established fibrosis [27]. Furthermore, it should be noted that although NDGA has been shown to preferentially inhibit lipoxygenase‐5 [64, 65, 66], its activity is not exclusive to lipoxygenases. Indeed, NDGA has also been shown to also block leukotriene B4 and it has potent antioxidative properties [67].

Dendritic cells (DCs) are antigen‐presenting cells known to influence T‐cell proliferation and plasticity. In this study, we showed that hAEC treatment significantly reduced DC infiltration (day 5) to the site of injury and this might subsequently have led to changes in T‐cell numbers that we saw on day 7. This study identified possible mechanisms of hAEC‐mediated regulation of T‐cell behavior and phenotype. Our coculture migration and proliferation assays found that T‐cell migration toward CCL17 was unaffected by the hAEC‐conditioned medium but was able to suppress T‐cell proliferation. To elucidate the consequence of hAEC‐derived LXA4 on T‐cell proliferation, we found that addition of NDGA mitigated hAEC suppression of T‐cell proliferation. This may be due to the inhibition of extracellular signal‐regulated kinase signaling, which is known to regulate T‐cell proliferation [59].

The bleomycin mouse model of pulmonary fibrosis has come under recent scrutiny [10]. IPF is a disease with a complex pathology, and simple anti‐inflammatory and antifibrotic approaches are not curative. Although the bleomycin model does not capture the full complexity of the disease, it does replicate the acute exacerbations of the disease [10] where proinflammatory cytokines predominate (e.g., TNF‐α, IL‐1β, IL‐6, TGF‐β, fibronectin, and procollagen‐1) [68]. Furthermore, at the early stages of inflammation, when PGE2 and LTB4 provoke neutrophil extravasation, LXA4 and its epimer 15‐epi‐LXA4 was shown to have a profound inhibitory effect on PMN infiltration in vivo [69]. Our focus on acute inflammatory events provided an opportunity to investigate the immune regulatory mechanism of hAECs during acute exacerbations. Immunological changes exerted by hAECs beyond this period were not examined and should be the subject of future research. Previous work showed 4 million hAECs given 24 hours after bleomycin challenge via intraperitoneal administration ameliorated lung fibrosis and restored lung function in bleomycin‐challenged mice irrespective of hAEC engraftment [4, 45]; thus, this model was selected for our study. Whether lower doses, repeated dosing, or varying the route of administration will improve acute inflammatory exacerbations and/or long‐term outcomes on fibrosis remains to be investigated. Assessing the efficacy of hAECs in a model of repeated bleomycin challenge will also likely be useful in terms of evaluating their therapeutic utility in intractable lung fibrosis.

## Conclusion

Our current study provides evidence that hAECs can alter neutrophil, macrophage, and T‐cell migration, phenotype, and behavior in resolving bleomycin‐induced lung injury, in part through LXA4. In the process of lung repair, hAECs promote resolution by upregulating macrophage phagocytosis, partly through LXA. It was highlighted at the 2012 National Institutes of Health, National Heart, Lung, and Blood Institute workshop that understanding the mechanism of action of stem cells is paramount for the advancement of cell‐based therapies [70]. Acumen of the biodynamics of cell therapy candidates in various disease or injury settings, therefore, will expedite the development of cell‐based therapies for lung disease. Additional studies on how these exogenously delivered stem cells and stem‐like cells aid other endogenous repair processes, such as their effects on stem/progenitor cell populations and extracellular matrices, during different phases of injury will be needed to best exploit their regenerative properties.

## Author Contributions

J.L.T.: study design, collection and/or assembly of data, data analysis and interpretation, manuscript writing, final approval of manuscript; Y.Z.T., R.M., S.T.C., and S.N.L.: collection and/or assembly of data, data analysis and interpretation, final approval of manuscript; J.C.M.: administrative support; final approval of manuscript; E.M.W.: study design, data analysis and interpretation, manuscript writing, final approval of manuscript; R.L.: study design, data analysis and interpretation, manuscript writing.

## Disclosure of Potential Conflicts of Interest

S.T.C. has compensated employment and intellectual property rights. E.M.W. holds a patent for use of amnion cells as a lung repair therapy. The other authors indicated no potential conflicts of interest.

## Supporting information

Supporting Information
