# Supplementary material for: Amnion Epithelial Cells Promote Lung Repair via Lipoxin A4
Source: Stem Cells Transl Med. 2016 Nov 7;6(4):1085–95. doi: 10.5966/sctm.2016-0077 (PMC5442827; doi:10.5966/sctm.2016-0077)
Supplement: Supplementary file 1 — Supporting Information [file SCT3-6-1085-s001.pdf]

Supplemental Figure – Lim et al.

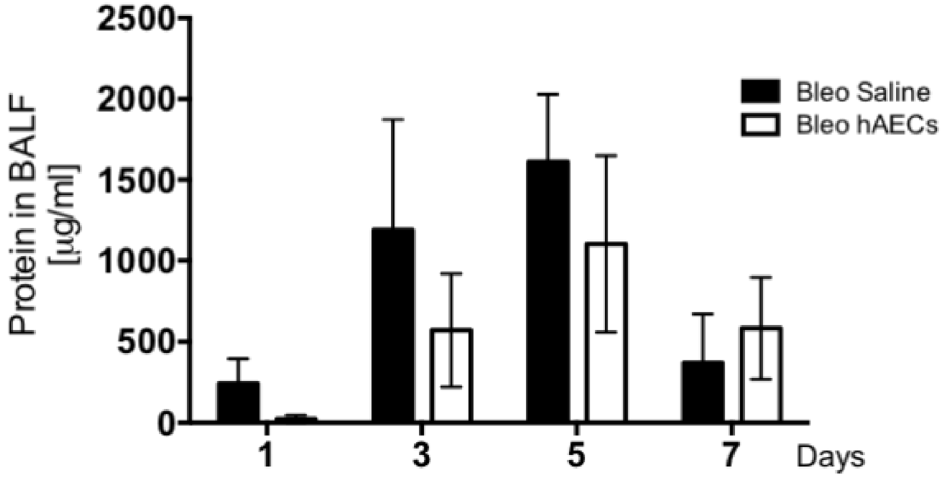

Figure S1. Protein concentration from bronchoalveolar lavage fluid collected showed no significant difference between both groups across all days (S1).
